# Enhanced Recovery after Surgery Applied to Pediatric Laparoscopic Cholecystectomy for Simple Cholelithiasis: Feasibility and Teaching Insights

**DOI:** 10.3390/children10121881

**Published:** 2023-11-30

**Authors:** Luca Pio, Berenice Tulelli, Liza Ali, Lucas Carvalho, Marc Chalhoub, Florence Julien-Marsollier, Arnaud Bonnard

**Affiliations:** 1 Department of General Pediatric Surgery and Urology, Robert Debré Children University Hospital, APHP, 75019 Paris, France; berenice.tulelli@chu-lyon.fr (B.T.); liza.ali@aphp.fr (L.A.); lucas.carvalho@aphp.fr (L.C.); mchalhoub@hopital-saint-joseph.fr (M.C.); arnaud.bonnard@aphp.fr (A.B.); 2 Paediatric Surgery Department, University Sorbonne Paris-Cité, 75006 Paris, France; 3 Department of Anesthesia, Intensive Care and Pain Management, Robert Debré Children University Hospital, APHP, 75019 Paris, France; florence.julien-marsollier@aphp.fr

**Keywords:** laparoscopic cholecystectomy, ERAS, pediatric surgery learning curve

## Abstract

Background: Same-day discharge after a cholecystectomy is a common practice in the adult population and has been demonstrated as safe and viable for children as well. However, there is a lack of comprehensive teaching models for pediatric cholecystectomy. Drawing inspiration from standardized outpatient procedures, this study aimed to assess the clinical outcomes and feasibility of teaching programs and an Enhanced Recovery After Surgery (ERAS) protocol following ambulatory laparoscopic cholecystectomy in pediatric patients. Methods: In 2015, an ERAS pathway for laparoscopic cholecystectomy (LC) was implemented, focusing on admission procedures, surgery timing, anesthetic choices, analgesia, postoperative feeding, mobilization, and pain assessment. Day-case surgery was not applicable for acute cholecystitis, choledochal lithiasis, sickle cell disease, and hereditary spherocytosis cases. The protocol was employed for a group of attending surgeons and fellows, as well as a group of residents under the supervision of experienced surgeons. A retrospective analysis was conducted to evaluate the feasibility and effectiveness of ambulatory cholecystectomy in children and its utilization in training pediatric surgical trainees. Results: Between 2015 and 2020, a total of 33 patients were included from a cohort of 162 children who underwent LC, with 15 children operated on by senior surgeons and 18 by young surgeons. The primary diagnoses were symptomatic gallbladder lithiasis (*n* = 32) and biliary dyskinesia (*n* = 1). The median age at the time of surgery was 11.3 years (interquartile range (IQR) 4.9–18), and the median duration of surgery was 54 min (IQR 13–145). One intraoperative complication occurred, involving gallbladder rupture and the dissemination of lithiasis into the peritoneal cavity. Three patients (9%) required an overnight stay, while no postoperative complications or readmissions within 30 days were observed. ERAS was successfully implemented in 30 patients (91%). No significant differences in surgical outcomes were noted between senior and young surgeons. At an average follow-up of 55 months, no long-term sequelae were identified. Conclusions: These findings align with the current trend of increasing use of outpatient laparoscopic cholecystectomy and underscore its feasibility in the pediatric population. The application of a structured ERAS protocol appears viable and practical for training the next generation of pediatric surgeons. Level of Evidence: Level III.

## 1. Introduction

Laparoscopic cholecystectomy (LC) has evolved over the years to become a standard and routine procedure in the realm of general surgery [1,2]. In the adult population, same-day discharge after surgery is a common and accepted practice, reflecting the safety and feasibility of this approach. The development of Enhanced Recovery After Surgery (ERAS) has played a significant role in achieving these positive outcomes. ERAS represents a multidisciplinary approach involving collaboration between surgeons, anesthesiologists, and nurses. This approach has consistently demonstrated significant improvements in surgical outcomes, including the promotion of early postoperative feeding and ambulation, a reduction in the need for postoperative painkillers, and the expeditious and safe discharge of patients [3,4].

As the success of standardized outpatient procedures has become evident, the implementation of ambulatory models in pediatric surgery is an exciting development [5]. This practice has previously been reported for various diseases, showing promise in improving patient experiences and overall healthcare delivery. Furthermore, recent years have seen the emergence of ERAS protocols specifically designed for LC in children, with studies reporting excellent results. These advancements underscore the importance of tailoring approaches to the unique needs and characteristics of the pediatric population [6,7,8,9,10,11].

The historical perspective of LC in children can be traced back to the pioneering work of Holcomb III in 1999. This early series emphasized LC as an effective and safe procedure for pediatric patients. Over time, it has evolved to become the gold standard, extending its applications even to the primary treatment of common bile duct stones [12,13]. The increased awareness of gallbladder disease as a potential cause of abdominal pain in children is partially attributed to changes in disease etiology and the more widespread use of ultrasonographic imaging techniques. These advances have improved the diagnosis and management of gallbladder conditions in pediatric patients [14].

Despite the substantial progress made in LC for children, it remains a relatively infrequent procedure for pediatric surgeons. While there have been numerous reports demonstrating the feasibility and safety of LC in children, there is still a significant gap in knowledge regarding the learning curve and teaching models in the pediatric field. This gap underscores the need for more comprehensive research and standardized approaches to pediatric LC [15,16].

A recent European survey on training in minimally invasive surgery highlighted the lack of experience among young pediatric surgeons in LC [17]. The learning curve and the development of teaching models are critical aspects of ensuring the successful and safe implementation of surgical procedures. In this context, Enhanced Recovery After Surgery (ERAS) protocols play a pivotal role in influencing adherence and improving patient outcomes [18]. However, in the field of pediatric surgery, there is a notable scarcity of available data concerning the integration of ERAS protocols into teaching programs and their impact on surgical education.

This study makes a valuable contribution to the existing literature by presenting the clinical outcomes following the utilization of the pediatric ambulatory laparoscopic cholecystectomy ERAS protocol. By doing so, it further delves into the successful application and feasibility of this protocol within a well-established teaching program.

This study aims to contribute to the existing literature by presenting clinical outcomes following the utilization of the pediatric ambulatory laparoscopic cholecystectomy ERAS protocol. This aspect of the study addresses a fundamental component of pediatric surgical education and practice, offering insights into how ERAS protocols can be effectively integrated into training and education programs for young surgeons.

## 2. Materials and Methods

In the quest to optimize the care pathway for laparoscopic cholecystectomy (LC) in pediatric patients, a carefully structured protocol was introduced in 2015. This protocol placed significant emphasis on various critical components of the surgical process, including the day of admission, the timing of surgery, choice of anesthetic agents, analgesia, postoperative feeding, mobilization, and pain scoring. It is important to note that this protocol was developed in close consultation with the local Institutional Review Board (IRB), with approval obtained under reference number 919 on 1 March 2019, ensuring adherence to ethical and safety guidelines.

### 2.1. Patient Selection and Inclusion Criteria

**Figure 1:**
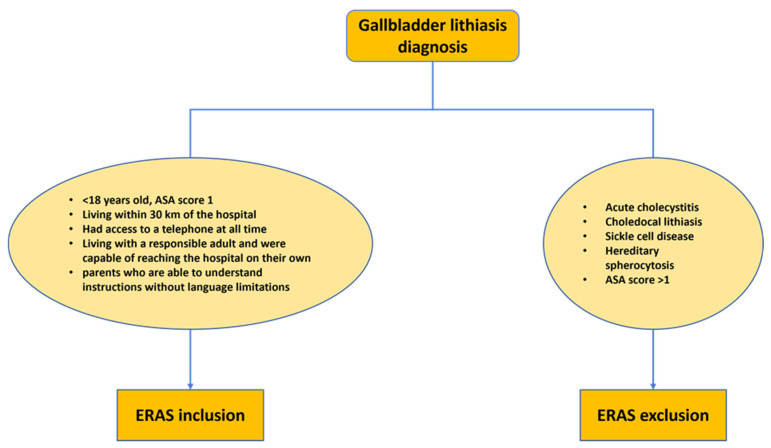
Enhanced recovery after surgery (ERAS) cholecystectomy protocol selection criteria. ASA: American Society of Anesthesiologists score.

To ensure the study’s relevance and validity, specific inclusion and exclusion criteria were established. Patients meeting the inclusion criteria had to be diagnosed with symptomatic uncomplicated gallbladder lithiasis. This clear diagnostic criterion allowed for the precise identification of the target population. In contrast, patients with acute cholecystitis, choledochal lithiasis, sickle cell disease, and hereditary spherocytosis were excluded from the study. These exclusion criteria aimed at maintaining a homogeneous study group focused on uncomplicated cases of gallbladder lithiasis (Figure 1).

### 2.2. Data Collection and Analysis

A retrospective analysis of demographic and clinical data was conducted for a five-year period, from June 2014 to June 2019. This comprehensive data collection span allowed for a robust examination of patient outcomes and experiences over time. All patients included in the study were systematically followed up with clinically to track their progress and identify any potential postoperative complications.

Comparative analyses between Groups 1 and 2 were conducted using the two-tailed chi-squared and Mann–Whitney U-tests. These statistical tests were chosen for their ability to provide rigorous and valid comparisons between the two groups. In line with established statistical practices, *p*-values less than 0.05 were considered statistically significant, ensuring that the observed differences were not due to chance.

### 2.3. Enhanced Recovery after Surgery (ERAS) Protocol

The ERAS protocol employed in this study incorporated several essential criteria for participant inclusion. These criteria were meticulously designed to ensure the safety and wellbeing of the pediatric patients. First and foremost, patients considered for inclusion had to be below 18 years of age. This age limit was set to focus on pediatric patients, given their distinct medical needs and considerations. ERAS protocol was applied to all children included in the study, regardless of the surgeon in charge of each patient.

Patients were further required to have an ASA (American Society of Anesthesiologists) score of 1, which signifies good overall health and minimal systemic illness. This criterion was crucial in selecting patients who were medically fit for the outpatient procedure, as patients with higher ASA scores may require more intensive medical attention.

Geographical proximity to the hospital was another key factor considered in patient selection. Patients needed to reside within a 30 km radius of the healthcare facility. This geographical limitation was established to ensure that individuals had convenient access to the hospital, thereby minimizing potential logistical challenges associated with distant travel.

Accessibility and effective communication were additional primary inclusion criteria. Parents or guardians of eligible patients were required to have the ability to comprehend and follow postoperative instructions effectively. This included understanding instructions without any language barriers, ensuring optimal postoperative care and adherence to guidelines. For minors, it was imperative that they lived with a responsible adult who could assist in their transportation to the hospital.

Lastly, parental understanding and comprehension of postoperative instructions were deemed crucial. Parents or guardians of eligible patients were required to be capable of comprehending these instructions without language barriers, ensuring proper postoperative care and compliance with prescribed guidelines. They were also expected to have continuous access to a telephone, enabling effective communication and rapid response in case of any queries or concerns.

### 2.4. Preoperative Evaluation and Preparation

Patients were seen in our patient clinic before surgery to explain the day-case procedure. A hepatobiliary blood test and ultrasound control were conducted in case of scheduled intervention if more than 3 months had passed since the diagnosis. This thorough evaluation helped identify any potential changes in the patient’s condition and excluded the possibility of lithiasis migration.

LC was scheduled as the first procedure on the outpatient clinic day list. This scheduling prioritization was aimed at minimizing potential delays and ensuring the efficient progression of the surgical day.

### 2.5. Anesthetic Management and Early Postoperative Care

Anesthesia for patients weighing less than 30 kg was started with gaseous anesthetic sevoflurane, then intravenous sufentanil, and administration of atracurium after orotracheal intubation. For patients > 30 kg, induction was performed with total intravenous anesthesia, using propofol (target 4 to 6 µg/mL), sufentanil (target 3 to 5 ng/mL), and atracurium (0.5 mg/kg). Early postoperative analgesia was performed by intraoperative paracetamol and ketoprofen injections. Local anesthesia by ropivacaine injections (0.2 mL/kg) in all trocar orifices was administered at the beginning of the procedure. In immediate postoperative unit care, nalbuphine 0.1 mg/kg via slow infusion was administered once.

### 2.6. Postoperative Feeding and Discharge Criteria

**Figure 2:**
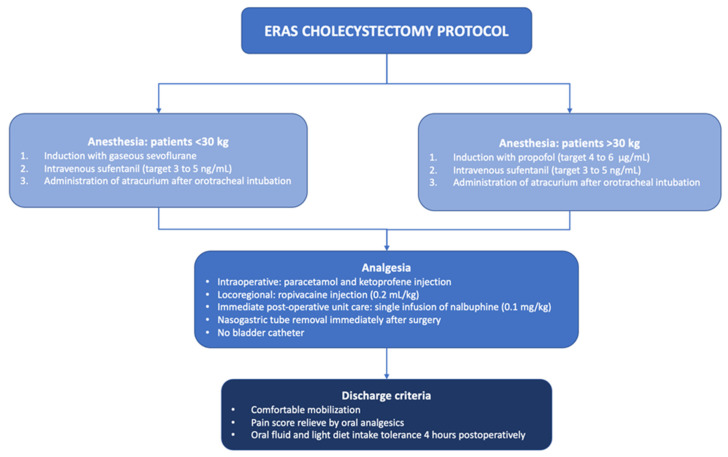
Enhanced recovery after surgery (ERAS) cholecystectomy protocol.

The postoperative feeding regime was not restricted, allowing patients to resume their regular diet as tolerated. Criteria for patient discharge included comfortable mobilization, pain scores within the range of levels I–II, and the ability to tolerate oral fluid and a light diet intake four hours postoperatively (Figure 2).

### 2.7. Postoperative Follow-Up and Support

On the first postoperative day, the ambulatory outpatient nurse was responsible for calling the patient’s home to ensure the child was in good condition and providing counselling and support for the family. Clinical follow-ups were subsequently performed.

### 2.8. Teaching Setting

LCs were performed by consultants or fellows (Group 1) and residents with at least a minimally invasive experience of 10 laparoscopic appendectomies and one LC in the ordinary elective program (Group 2). Trainees managed the entire procedure under a fellow’s supervision, including patient positioning and MIS setting, and handled the principal laparoscopic gestures, including dissection, hemostasis control, and intracorporeal knotting. The laparoscopic four-port technique provided a 30° 10 mm camera umbilical port and 3 to 5 mm secondary ports. CO_2_ insufflation pressure of 10 mmHg and low insufflation rate of 1.5–2 L/min were used. Retrograde cholecystectomy was performed, with cystic artery bipolar or monopolar hook coagulation and cystic duct ligature with two intracorporeal 4/0 absorbable knots. Gallbladder was removed through the umbilical trocar incision. ERAS protocol was equally applied to both groups.

## 3. Results

During the study period from 2015 to 2020, our institution conducted a comprehensive investigation involving a total of 33 pediatric patients who underwent outpatient laparoscopic cholecystectomy (LC). Among this group, there were 15 females and 18 males. It is important to note that these patients were part of a larger cohort of 162 children who had undergone LC at our institution during the same timeframe. However, for the purposes of this study, 129 patients were excluded from the analysis. This exclusion was primarily due to specific medical conditions necessitating specialized perioperative and postoperative protocols. These excluded conditions included spherocytosis, a condition that requires dedicated care and specific surgical procedures, such as a splenectomy. Additionally, cases involving common bile duct cholelithiasis were excluded due to their complex nature.

The patients included in this study primarily presented with symptoms of recurrent abdominal pain or biliary colic. Upon clinical evaluation, their diagnoses were categorized into two primary groups: symptomatic gallbladder lithiasis (*n* = 32) and biliary dyskinesia (*n* = 1).

To comprehensively assess predisposing factors for biliary lithiasis in the study cohort, all patients underwent thorough screening and evaluation. This screening included various tests, such as hemoglobin electrophoresis, lipid profile assessment, evaluation for signs of hemolysis, and comprehensive liver function tests. Among the screened patients, a small proportion (*n* = 3) had comorbidities, including trisomy 21, Crohn’s disease, and multiple endocrine neoplasia type B syndrome. These comorbidities added complexity to the overall patient management but were considered within the study’s scope.

The implementation of anesthesiology management was crucial for ensuring the safety and success of the outpatient LC procedures. In this study, anesthesia management was meticulously aligned with the Enhanced Recovery After Surgery (ERAS) protocol. Significantly, this protocol was successfully applied to all patients, resulting in a complete absence of perioperative complications.

The surgical procedures were distributed among two distinct groups of surgeons (Table 1). In Group 1, the surgical team consisted of experienced consultants and fellows, who brought a wealth of surgical expertise to the procedures. In contrast, Group 2 comprised residents with a specified level of laparoscopic experience. This distribution allowed for a comparative assessment of outcomes based on the experience and skill level of the surgical team.

The mean age of the patients at the time of surgery was 11.3 years, with individual patient ages ranging from 4.9 to 18 years. This wide age range enabled the study to assess the outcomes of laparoscopic cholecystectomy across various pediatric age groups. The mean operative time for the procedures was approximately 54 min, with individual surgical procedures varying in duration, ranging from 13 to 145 min. Importantly, a comparative analysis between Group 1 and Group 2 did not reveal significant differences in terms of patient age and the presence of comorbidities.

During the study period, only one intraoperative complication (3%) occurred in Group 2. This complication was characterized by an intraoperative gallbladder perforation, resulting in bile dissemination within the peritoneal cavity. Importantly, this complication did not necessitate postoperative drainage or more invasive interventions. Remarkably, in this study, no conversions from laparoscopy to laparotomy were observed, highlighting the overall success and safety of the minimally invasive approach.

The ERAS protocol was effectively implemented in 91% of the cases, accounting for 30 patients. This successful application of the ERAS protocol enabled the direct discharge of these patients from the recovery room. In contrast, the remaining 9% of patients (three individuals) required an overnight stay, primarily for enhanced pain control or observation following the aforementioned intraoperative complication.

Postoperatively, all discharged patients were followed up with by the ambulatory outpatient nurse within the first postoperative day. This follow-up process was instrumental in ensuring the effective monitoring of their recovery. There were no postoperative complications or readmissions during the course of the study.

A median clinical follow-up period of 55 months, ranging from 6 to 60 months, was conducted to assess the long-term outcomes of the pediatric patients who underwent surgery. No sequelae associated with lithiasis or the surgical procedures were observed.

## 4. Discussion

Cholecystectomy is a routine and standardized procedure in general adult surgery. However, in pediatric surgery, the management of patients and surgical training remain topics of ongoing debate. A recent collaborative survey conducted jointly by the International Pediatric Endosurgery Group and the European Paediatric Surgeons’ Association members has shed light on a concerning lack of education and implementation of Enhanced Recovery After Surgery (ERAS) protocols for minimally invasive surgery (MIS) within the pediatric surgery community [19]. Furthermore, a recent emphasis has been placed on the inadequate exposure to MIS among European pediatric surgery trainees [17]. Moreover, in recent years, the SARS-COVID-19 pandemic highlighted the need for structured training programs [20]. Given these challenges, the present study stands out as a unique endeavor that comprehensively analyzes both the implementation of ERAS and training aspects within pediatric MIS. Our findings underscore the safety and effectiveness of ERAS in laparoscopic cholecystectomy (LC) for children. Additionally, our study suggests that ERAS protocols can be effectively applied in a training context, as no discernible clinical outcome differences were observed between patients operated on by experienced senior surgeons and those supervised by residents.

Gallbladder diseases are relatively rare in children, especially when compared to adults. The increasing epidemic of childhood obesity, where approximately one third of children are overweight or obese, along with an increased incidence of functional gallbladder disorders, is thought to account for most pediatric cholecystectomies performed [21].

Although laparoscopic cholecystectomy (LC) has become widely adopted, leading to numerous high-quality scientific studies reporting its feasibility and safety in day surgery, it is less frequently performed in the pediatric field. As a result, pediatric LC is subject to reduced surgical expertise and confidence.

The analysis of a large multicenter dataset from the National Surgical Quality Improvement Program has demonstrated that children without significant associated comorbidities can safely undergo outpatient laparoscopic cholecystectomy [22].

The growing importance of ERAS in pediatric surgery has gained prominence over the years. Its well-established safety and efficacy in the current context can significantly impact economics by enabling families to return to work more swiftly [23,24,25]. Even though minimally invasive surgery has historically been a concern in pediatric outpatient procedures, recent studies have shown the feasibility of implementing ERAS in the pediatric population, resulting in a noteworthy rise in the rate of same-day discharges, ranging from 77.2% to 78% after the implementation of their ambulatory protocols [7,8]. Interestingly, our study achieved an impressive 91% ERAS adherence, even without the use of opioids, as observed in previous research. This outcome could be attributed to the careful patient selection in our protocol, which specifically targeted individuals with an ASA score of 1, no language barriers, and proximity to the hospital. Consistent with the growing trend of ERAS in pediatric surgery, our results show the feasibility and safe application of this approach for pediatric gallbladder lithiasis, resulting in a low complication rate and outpatient discharge rates comparable to the current literature [11,12,13,14,15,16].

ERAS cholecystectomy was performed for uncomplicated lithiasis, focusing on the highest and relatively more straightforward volume cases to facilitate the hepatobiliary learning curve in pediatric surgery.

While the learning curve of young pediatric surgeons has been somewhat overlooked in the literature [26,27], it has been extensively studied in other surgical fields such as general surgery and orthopedics, yielding excellent mentoring results with an adequate case volume exposure [28].

The assessment of competence in laparoscopic cholecystectomy in trainee populations exhibits substantial heterogeneity in the literature. A recent study reported a median caseload of 95 procedures required to achieve adequate competence in laparoscopic cholecystectomy for general surgeons [29]. However, such volumes are challenging to attain in pediatric surgery within a single institution, even throughout the entirety of a residency.

New techniques, such as transvaginal, single-port, and robotic cholecystectomies, have reported different caseload volumes to achieve an adequate level of competence with those techniques, with 15, 8, and 48 procedures, respectively, needed to achieve adequate surgical competence [30,31,32].

Compared to adult general surgeons, pediatric residents and young fellows typically encounter fewer hepatobiliary procedures due to the lower incidence of cases, and some countries do not provide adult general surgery training during internships [26]. The establishment of an ERAS protocol is of paramount importance in ensuring consistent exposure to hepatobiliary procedures, given the continuous generational turnover in the pediatric surgeons’ residency.

Our study underscores that ERAS can be beneficial and safe for the surgical learning of hepatobiliary procedures by young pediatric surgeons. It yields favorable outcomes without significantly increased complications when compared to senior surgeons. Moreover, the successful application of the ERAS protocol in a teaching hospital not only validates its safety and efficacy but also supports the integration of ERAS principles into the training and education of future pediatric surgeons. It sets a precedent for incorporating ERAS as a standard approach in teaching institutions, thereby influencing and shaping the practices and training of the next generation of surgical professionals. Furthermore, ERAS pathway education has been described as one of the most important factors with a favorable impact on surgical outcomes; thus, involvement of young trainees should be included in this model of patient care [18]. The incorporation of teaching programs for day-case procedures facilitated by the implementation of ERAS in LC is another noteworthy discovery in the realm of surgical education. While there is a lack of comprehensive data in pediatric surgery, studies focusing on adult training have revealed an inconsistent level of exposure to day-case procedures and a high degree of dissatisfaction among surgical trainees [33]. In our study, the consistent performance of LC as an outpatient procedure, thanks to the ERAS protocol, has significantly increased the active involvement of trainees in the day surgery schedule. This finding holds significant implications for the continuous improvement of surgical education, ultimately benefiting the care and outcomes of pediatric surgical patients.

Our study highlighted that the introduction of a new surgical pattern of care can be feasible and safe for children with the direct involvement of trainees in their surgical management.

While the primary limitation of our study lies in the relatively small cohort of nonrandomized patients, the use of a standardized teaching model has proven effective in achieving favorable surgical outcomes, despite the limited number of pediatric cases. Another potential source of bias stems from the variability in the number of surgeons in both groups.

These findings align with the increasing trend of utilizing outpatient laparoscopic cholecystectomy over time and confirm recent observations regarding the feasibility of cholecystectomy in the pediatric population with a well-coordinated joint surgical and anesthesiologist protocol. However, given the limited number of pediatric LC cases, larger series are required to confirm our results concerning the effectiveness of ERAS in managing childhood cholelithiasis and its impact on surgical training.

## 5. Conclusions

Applying an ERAS protocol for pediatric LC in a teaching setting hospital appears feasible and safe, providing the same results in terms of postoperative complications for patients operated on by residents under supervision by a senior surgeon. The positive outcomes observed through the implementation of an ERAS protocol in pediatric LC within a teaching hospital setting reaffirm its suitability and safety. It also emphasizes the potential for enhancing surgical training and education through the systematic integration of ERAS principles, ultimately elevating the standard of care and ensuring a more informed and proficient surgical workforce.

## Figures and Tables

**Table 1:** General demographic and surgical data of all patients operated on from 2015 until 2020. Group 1: consultant/fellow pediatric surgeons; Group 2: residents in pediatric surgery. IQR: interquartile range; * chi-square test; ^#^ Mann–Whitney U-test.

	Patients (*n* = 33)	Group 1 (*n* = 15)	Group 2 (*n* = 18)	*p* Value
Gender (n,%)				
Male	18 (54.5%)	7 (46.7%)	11 (61.1%)	0.63 *
Female	15 (45.5%)	8 (53.3%)	7 (38.9%)	
Median age during the operation (years, IQR)	11.3 (4.9–18)	11.3 (7.7–16)	12.2 (4.9–18.6)	0.46 ^#^
Comorbidities (n,%)	3 (10%)	2 (13.3%)	1 (5.5%)	0.44 *
Median surgical time (minutes, IQR)	54 (13–145)	40 (13–95)	53.5 (31–145)	0.11 ^#^
ERAS effectiveness (n,%)	30/33 (91%)	15 (100%)	15 (83%)	0.29 *
Intraoperative complication rate (n,%)	1 (3%)	-	1 (5.5%)	0.35 *
Postoperative complication rate (n,%)	-	-	-	-
30 days readmissions (n,%)	-	-	-	-
Median follow-up duration (months, IQR)	55 (6–60)	40 (6–60)	30 (12–60)	0.20 ^#^

## Data Availability

Data are contained within the article.
